# The Role of Substance P in Ischaemic Brain Injury 

**DOI:** 10.3390/brainsci3010123

**Published:** 2013-01-30

**Authors:** Renée J. Turner, Robert Vink

**Affiliations:** Adelaide Centre for Neuroscience Research, School of Medical Sciences, The University of Adelaide, Adelaide 5005, Australia; E-Mail: robert.vink@adelaide.edu.au

**Keywords:** substance P, neuropeptides, neurogenic inflammation, cerebral oedema, stroke, tachykinin, blood-brain barrier, cerebral ischaemia

## Abstract

Stroke is a leading cause of death, disability and dementia worldwide. Despite extensive pre-clinical investigation, few therapeutic treatment options are available to patients, meaning that death, severe disability and the requirement for long-term rehabilitation are common outcomes. Cell loss and tissue injury following stroke occurs through a number of diverse secondary injury pathways, whose delayed nature provides an opportunity for pharmacological intervention. Amongst these secondary injury factors, increased blood-brain barrier permeability and cerebral oedema are well-documented complications of cerebral ischaemia, whose severity has been shown to be associated with final outcome. Whilst the mechanisms of increased blood-brain barrier permeability and cerebral oedema are largely unknown, recent evidence suggests that the neuropeptide substance P (SP) plays a central role. The aim of this review is to examine the role of SP in ischaemic stroke and report on the potential utility of NK1 tachykinin receptor antagonists as therapeutic agents.

## 1. Introduction

Stroke is a major health problem in western nations and is a leading cause of morbidity, mortality and dementia. Specifically, each year a staggering 15 million people worldwide will suffer a stroke, of which 5 million will die and 5 million will be left permanently disabled. The social and economic costs of stroke are consequently enormous. In Australia alone, the cost of hospitalisation, treatment and rehabilitation of stroke patients is estimated at $1.2–1.7 billion per year [1]. At present, thrombolysis with tissue plasminogen activator within 4.5 h of symptom onset is the only approved stroke therapy [2] but is only received by some 5%–15% of stroke patients. As such, novel therapies that can limit or reverse ischaemic injury are urgently required. 

## 2. Secondary Injury

**Figure 1:**
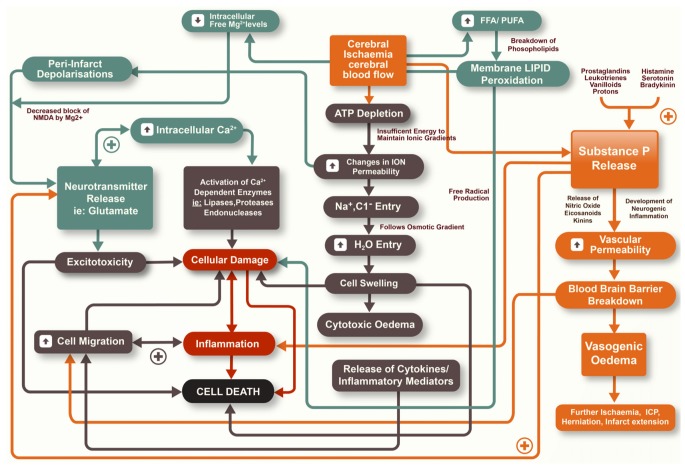
Secondary injury pathways that occur following ischaemic stroke.

Ischaemic stroke is the result of an obstruction to the brain vasculature, thereby restricting the supply of blood that contains vital oxygen and substrates for neurons. If blood flow is not rapidly restored, death of cells may result with associated long-term functional deficits [3]. Whilst restoration of blood flow is seen as a priority in both reducing the degree of tissue injury and preserving neurological function, it is now accepted that secondary injury mechanisms continue to evolve after stroke and also contribute to the size of the infarct [4]. The infarction can be considered as being made up of two components, the infarct core and the surrounding penumbral tissue [5]. The infarct core is widely considered to be irreversibly damaged during ischaemic stroke with cell death occurring rapidly within this region. In the penumbral tissue, however, there is less restricted blood flow and therefore an opportunity for neuronal tissue to survive the insult. Nonetheless, cell death may continue to occur here as a result of secondary biochemical and physiological mechanisms that manifest over the hours to days following stroke [4,5]. There is a diverse array of secondary injury processes that contribute to injury and cell loss, including excitotoxicity, oxidative stress, inflammation, apoptosis, increased vascular permeability and cerebral oedema, amongst others (Figure 1) [6]. Given the delayed nature of secondary injury an opportunity therefore exists for pharmacological intervention to limit tissue damage and cell death. Accordingly, much research has focused on the characterization of secondary injury pathways so as to develop therapies that reduce or ameliorate such pathways.

### 2.1. Blood-Brain Barrier (BBB)

The blood-brain barrier (BBB) is a highly selective barrier that serves to regulate the entry of blood-borne substances, some of which may damage the fragile brain extracellular environment [7]. It is made up of a complex cellular system of cells of the cerebral capillaries and post-capillary venules resting on the basal lamina [6]. The BBB facilitates a constant supply of nutrients, preserves ion homeostasis within the brain microenvironment, and protects against noxious chemicals, variations in blood composition and the breakdown of concentration gradients. The gate function of the BBB is provided by the tight and adherin junctions, a complex network of transmembrane and cytosolic proteins [7]. However, in conditions of ischaemia, there is a loss of BBB integrity such that alterations in vascular permeability and basal lamina structure occur [8,9]. Deprivation of blood flow to downstream microvasculature activates a cascade of events, including activation of coagulation factors, disruption of the extracellular matrix and increased capillary permeability [10,11]. Endothelial dysfunction occurs and leads to early permeability increases that cause extravasation of plasma components and oedema formation [7,10]. In addition, loss of basal lamina integrity causes red blood cell leakage and the development of haemorrhagic transformation; inflammatory cells, cytokines, proteases and free radicals have all been linked to such damage [6,12]. 

The exact mechanism by which ischaemia disrupts the BBB is debatable; however acute hypertension, hyperosmolar solutions, inflammation and more recently the matrix metalloproteinase family, have all been implicated [7]. The exact time course of BBB disruption following stroke also remains a contentious issue, although experimental studies have demonstrated a biphasic opening of the BBB [9,11,13], with early changes in BBB permeability observed at 2–3 h after the onset of vascular occlusion, while a second and delayed opening is typically observed at 24–48 h, the latter being associated with more intense blood vessel damage [11]. 

### 2.2. Cerebral Oedema

Of all the secondary injury factors, cerebral oedema, defined as an abnormal accumulation of fluid within the brain, is of particular concern given its association with increased mortality and morbidity after stroke [14,15]. Klatzo and colleagues [16] were the first to classify oedema into two broad categories based upon the integrity of the BBB: vasogenic and cytotoxic oedema [12]. The type and severity of cerebral oedema may be influenced by the duration and severity of ischaemia and reperfusion status, amongst other factors, and may also differ between the core and the penumbra of the stroke lesion.

Cytotoxic oedema is an intracellular oedema that occurs secondary to cellular injury and represents a shift of water from the extracellular compartment to the intracellular compartment, accompanied by shrinkage of the extracellular space [6]. It occurs independently of alterations in the BBB and appears to be more prominent in the grey matter [15]. Failure of the Na^+^/K^+^ ATPase in regions of energy failure and subsequent loss of ion homeostasis, leading to influx of water into cells, is central to the development of cytotoxic oedema [11,17,18]. Conversely, vasogenic oedema is more prevalent in the white matter [15] and involves the escape of proteins from the vasculature in the setting of BBB disruption and injury to cerebral blood vessels [11]. Protein accumulation in the brain extracellular space causes an osmotic increase at the site of injury and the subsequent movement of water down its osmotic gradient [11]. There is a strong correlation between extravasation of proteins into the extracellular space and the development of vasogenic oedema [9,13]. Vasogenic oedema is of particular importance not only because it increases brain volume, but also because in the setting of vascular recanalisation it increases risk of haemorrhagic transformation from damaged blood vessels and excess fluid accumulation [10].

Cerebral oedema is a major cause of clinical deterioration within the first 24 h, the leading cause of death within the first week, and is a predictor of poor outcome following stroke [14]. Clinical studies report that it is maximal between 1 and 3 days following stroke [15], whilst experimental studies report its presence as early as 15 min after the onset of vascular occlusion [19]. Cerebral oedema can lead to an increase in intracranial pressure (ICP), the sequelae of which include reduced cerebral blood flow, further ischaemia and infarct extension, deformation and herniation of the brain tissue, and in severe cases, death [10,14,15,20,21]. With the mortality of malignant cerebral oedema approaching 80% [15,22], the importance of reducing cerebral oedema and the resultant rise in ICP is now widely recognised as a major clinical management target. Conventional treatments seek to reduce brain swelling and ICP though administration of hyperosmotic agents and barbiturates, induction of hyperventilation or hypothermia, and surgical interventions such as cerebrospinal fluid (CSF) drainage, or in severe cases, decompressive craniectomy [14]. With respect to patient morbidity and mortality such treatment regimes have proven somewhat ineffective, mainly because they do not address the specific mechanisms that are associated with the genesis of oedema in cerebral ischaemia. Recent studies have identified substance P (SP) release as a feature of acute injury to the brain and have delineated a critical role for SP in increased BBB permeability and the development of vasogenic oedema.

## 3. Substance P

SP is an 11 amino acid peptide that is a member of the tachykinin family, so named for their fast-acting properties [23], which also includes neurokinin A (NKA), neurokinin B (NKB), neuropeptide K (NPK) and neuropeptide γ (NPγ), amongst others. It was first isolated by von Euler and Gaddum in the 1930’s as a crude extract from equine brain and gut that demonstrated potent hypotensive and smooth muscle contractile properties. It was subsequently named substance “P” for the powder from which it was extracted [24]. Initially, SP was identified in high concentrations in the dorsal root ganglia of the spinal cord, which drove speculation that it functioned as a neuronal sensory transmitter in the transmission of pain signals. It is now known that SP is released from both central and peripheral endings of primary afferent neurons where it functions as a neurotransmitter [23,25].

### 3.1. Synthesis

SP, along with other tachykinins, is produced from the preprotachykinin (PPT) A and B genes. Alternate splicing of the PPTA gene yields the β- and γ-transcripts giving rise to SP, NKA, NPK and NKγ, whereas the α- and δ- transcripts produce SP only. The PPTB gene only encodes for NKB. SP synthesis occurs at the cell body ribosomes where it is then packaged into vesicles and axonally transported to the terminal endings for final enzymatic processing [26]. Precursor proteins are stored in secretory granules along with processing enzymes for post-translational modifications and release of the active peptide [27,28]. The biologically active peptide is then stored in large, dense vesicles ready for release. Under normal conditions substantial amounts of SP are synthesised and stored within neurons [26]. However, activation or damage of these neurons results in the rapid release of SP and other neuropeptides [27]. 

### 3.2. Localisation

SP is widely distributed throughout the central and peripheral nervous systems, with α-PPTA transcripts more abundant within the brain and β-PPTA transcripts more abundant in peripheral tissues. The main feature of SP immunoreactivity is co-localisation with other classical transmitters such as serotonin and glutamate, and other neuropeptides such as calcitonin gene-related peptide (CGRP) and NKA [26,29]. Specifically, in the brain SP immunoreactivity has been demonstrated in the rhinencephalon, telencephalon, basal ganglia, hippocampus, amygdala, septal areas, diencephalon, hypothalamus, mesencephalon, metencephalon, pons, myelencephalon and spinal cord. In peripheral tissues, SP and other sensory neuropeptides are distributed throughout the gut, respiratory system, urinary system, immune system, blood and blood vessels [30].

SP is localised in capsaicin sensitive neurons and is released from central and peripheral endings of primary afferent neurons in response to various noxious stimuli [27,31]. Capsaicin itself causes the release of neuropeptides from these sensory nerve fibres to the point of depletion, hence being referred to as “capsaicin-sensitive” neurons. However, chronic exposure or high concentrations of capsaicin leads to permanent depletion [32], thereby blocking the effects of neuropeptides in the genesis of neurogenic inflammation and resultant oedema. Accordingly, acute and chronic capsaicin treatment is a useful tool to study the effects of neuropeptides in various physiological and pathological settings.

### 3.3. Metabolism

Once released, SP may be cleared and inactivated by many different proteolytic enzymes including neutral endopeptidase (NEP) [33,34], angiotensin-converting enzyme (ACE) [33,35,36], SP-degrading enzyme [33,37], post-proline endopeptidase [33,38], cathepsin-D [39], cathepsin-E [40], SP-hydrolysing enzyme, aminopeptidase P and dipeptidyl aminopeptidase IV [33]. All of these enzymes have the capacity to degrade SP *in vitro*, however it is likely that ACE and NEP are primarily involved in the cleavage of SP *in vivo* due to their cellular location [27]. Both of these enzymes catalyse the degradation of the hydrolytic bonds of SP, rendering it inactive without the carboxyl terminus required to bind to its receptor [26]. Specifically, NEP has been shown to degrade SP within the brain [41], spinal cord [42] and peripheral tissues [27] whereas ACE has been shown to degrade SP in plasma, CSF and brain, in particular the substania nigra [43].

### 3.4. Receptors

The biological actions of SP are mediated through its binding at tachykinin NK receptors which is a member of the rhodopsin family of 7-transmembrane G-protein coupled receptors [44]. Currently, 3 mammalian tachykinin receptors have been identified, the NK1, NK2 and NK3 receptors [45]. The tachykinins themselves share some structural homology, a carboxyl terminal sequence that reflects their common biological action. Given this, some cross-reactivity amongst tachykinin receptors exists [46] with each of the tachykinins acting on all receptor types, however with varying affinities depending upon receptor availability and neuropeptide concentration. Under normal conditions SP has a high affinity for the NK1 receptor, NKA for the NK2 receptor and NKB for the NK3 receptor [47,48]. With respect to the NK1 receptor, it is a 403 amino acid residue protein that is highly conserved with only discrete variations amongst species. An NK1 autoreceptor has also been characterised purported to be involved in the regulation of SP release [49,50,51,52]. NK1 receptors are found in their highest levels in the caudate putamen and superior colliculus, however they are also found in low to moderate levels in the inferior colliculus, olfactory bulb, hypothalamus, cerebral cortex, septum, striatum, mesencephalon and dorsal horn of the spinal cord [53]. 

Tachykinins are released in response to Ca^2+^-dependent depolarisation of neurons, induced by a variety of stimuli including electrical stimulation, pH changes and ligand binding to their receptors, including capsaicin [28,30]. Once released, SP may have direct post-synaptic actions as a neurotransmitter, modulatory function at post-synaptic sites or other functions on non-neuronal targets [28]. Transduction of the SP signal occurs through the action of G proteins associated with the intracellular domain of the NK1 receptor. The stimulation of G proteins produces an elevation in cAMP as a secondary messenger, which through a cascade of events leads to the regulation of ion channels, enzyme activity, and changes in gene expression. Although normally confined to the cell membrane, the NK1-SP complex is rapidly internalised following SP binding. SP is then removed by endosomal acidification and targeted by the lysosomes for degradation, whilst the NK1 receptor is recycled to the cell membrane [28].

### 3.5. Functions

Tachykinins are involved in a diverse array of distinct biological processes such as plasma protein extravasation, vasodilation, smooth muscle contraction and relaxation, airway contraction, transmission of nociceptive responses, salivary secretion, inflammation as well as higher functions including memory formation and reinforcement [47,54]. In terms of pathophysiology, SP has been implicated in asthma, inflammatory bowel disease, pain, psoriasis, anxiety, migraine, emesis and movement disorders as well as neurological and psychiatric disorders such as psychosis, stroke, migraine and pain. Increased SP levels have also been associated with painful conditions such as peripheral neuropathy and osteoarthritis. Intracerebroventricular injection of SP in rats results in a diverse array of effects including increased blood pressure and heart rate, increased hindlimb rearing behaviour, scratching, skin biting and grooming. Injection of SP into the lateral septum induces clear aversive behaviour including freezing and jumping followed by darting behaviour in the elevated plus maze [55]. In guinea pigs, SP release in the basolateral amygdala elicits distress vocalisations, which can be inhibited by the NK1 tachykinin receptors antagonist L760 735 [56]. Post-synaptic dorsal column neurons do not the express NK1 tachykinin receptor under control conditions, however in visceral inflammation, de novo expression of the NK1 tachykinin receptor occurs, thereby allowing the activation of such neurons by SP [57].

### 3.6. Trigeminovascular System

Cerebral blood vessels are highly innervated with a combination of sympathetic, parasympathetic and trigeminal somatic nerve fibres, all of which play important roles in cerebrovascular regulation [58]. The trigeminal component of this innervation is commonly referred to as the trigeminovascular system, which has been shown to be involved in the transmission of pain sensation. The perivascular endings of these trigeminovascular fibres contain SP [59], CGRP [60,61], NKA, nitric oxide [62] and amylin [63].

## 4. Neurogenic Inflammation

The concept of neurogenic inflammation was first documented by Bayliss in 1901 [64], who reported vasodilation of the lower limbs in response to dorsal root ganglia stimulation. The definition of neurogenic inflammation has now evolved to encompass a painful local inflammatory response characterised by vasodilation, increased vascular permeability, mast cell degranulation and the release of neuropeptides including SP and CGRP [30,65]. There are also tissue specific responses including smooth muscle contraction/relaxation in the bladder and ionotropic/chronotropic effects on the heart and bronchoconstriction in the airways, amongst others [47,66]. Neurogenic inflammation has been demonstrated in tissue receiving trigeminal innervation such as oral, nasal, facial and ocular tissue, and may be stimulated by many agents including prostanoids, leukotrienes, histamine and serotonin, in addition to changes in the extracellular environment such as decreased pH, increased osmolarity, heat, inflammatory conditions and tissue injury [27,67]. 

The changes in blood vessel size and permeability that occur with neurogenic inflammation lead to oedema formation within the tissue [9,13]. Perhaps the most important factor in this response is SP, having been identified as the most potent initiator of neurogenic inflammation [25,68]. However, it has not been established that SP is the sole peptide with the direct/indirect ability to cause vasodilation and plasma extravasation [30]. Neurogenic inflammation leads to increases in both PPT mRNA [27] and NK1 receptor mRNA [69]. Oedema and plasma extravasation in response to SP are also associated with the release of CGRP, release of histamine and serotonin from mast cells and the release of prostanoids and nitric oxide (NO) [47,66]. Some of the effects of SP are related to the ability of the peptide to activate neutrophils and thereby produce hydrogen peroxide, superoxide anion and NO. The pro-inflammatory effects of tachykinins are also related to activation of nuclear transcription factors such as nuclear factor κB [54,70] that increases cytokine levels [70,71].

### 4.1. Neurogenic Inflammation in the Peripheral Nervous System

The release of neuropeptides, and in particular SP, has long been known to initiate neurogenic inflammation in peripheral tissues such as the skin and trachea [30,47]. For example, arterial administration of SP leads to vasodilation and plasma extravasation. Alves and colleagues found that SP, NKA or NKB injection increase in rat paw oedema, as indicated by increased paw volume [72], and that other NK1, NK2 and NK3 receptor agonists similarly increase paw oedema. Consistent with this, NK1, NK2 and NK3 tachykinin receptor antagonists inhibit oedema caused by SP, NKA and NKB, respectively, in a dose dependent manner. Such findings confirm that SP, NKA and NKB play a role in the control of plasma extravasation and oedema formation and it is likely that all three receptor subtypes account for the pro-inflammatory response observed [72]. However, studies in knockout mice have shown that loss of the NK1 tachykinin receptor results in decreased plasma extravasation within peripheral tissues. In addition, there was a loss of the chemoattractant influence of SP, blunted noxious chemical signalling, decreased anxiety and blunting of response to danger. Studies examining neurogenic inflammation in the skin of NK1 tachykinin receptor knockout mice have also shown that they are unable to produce oedema, even though application of SP produces plasma extravasation and oedema formation in a dose-dependent manner in wild-type mice [31]. In guinea pig skin, SP causes oedema formation and white blood cell accumulation, both of which are inhibited by co-injection of the NK1 tachykinin receptor antagonist RP 67580 [54]. In mouse ear skin, SP, NKA and NKB all cause oedema formation. While all of these neuropeptides play a role in controlling plasma extravasation and oedema formation, SP is the major contributor. NK1 tachykinin receptor antagonists are able to inhibit oedematous responses caused by the various pro-inflammatory agents.

### 4.2. Neurogenic Inflammation in the Central Nervous System

In contrast to classical inflammation the concept of neurogenic inflammation in the brain has until very recently remained largely unexplored. Chemical, electrical or immunological stimulation, or treatment with capsaicin, was found to elicit a neurogenic inflammatory response in the dura mater but not the pia or cerebral cortex [73]. Similarly, intravenous administration of SP to rats has been shown to cause a significant increase in plasma extravasation in the dura mater, an effect abolished by pre-treatment with an NK1 tachykinin antagonist [74]. It was previously proposed that the activation of NK1 tachykinin receptors on vascular endothelium may contribute to cerebral oedema [75]. Subsequent studies in a rat diffuse traumatic brain injury (TBI) model have directly confirmed a role for SP and neurogenic inflammation in BBB dysfunction and the genesis of vasogenic oedema [76,77,78]. Taken together, these findings indicate that the release of SP may be central to changes in BBB permeability following cerebral insults. More recently, the transient receptor potential V1 (TRPV1), or the capsaicin receptor, has gained attention as an effector of neuronal injury, largely because activation of the TRPV1 receptor initiates neurogenic inflammation. Indeed, there is a high degree of localisation of the TRPV1 receptor with SP and CGRP [79] and TRPV1 activation leads to an increase in BBB permeability, an effect that is blocked by the TRPV1 antagonist capsazepine [80]. Clearly, TRPV1 plays a role in BBB dysfunction in the setting of acute injury to the brain, most likely as a facilitator of neurogenic inflammation.

### 4.3. Traumatic Brain Injury

Our group was the first to extend the concept of neurogenic inflammation to the brain in studies of TBI, demonstrating that SP release was a ubiquitous feature of acute injury to the brain and was associated with marked increases in BBB permeability and the development of vasogenic oedema and persistent functional deficits [81]. Specifically, following diffuse TBI in rats, an increase in SP immunoreactivity within the injured brain tissue was particularly evident in the perivascular tissue and was shown to co-localise with exogenously administered Evan’s Blue (EB), supporting a role for SP in increased vascular permeability following trauma [81]. Using the same model, animals chronically pre-treated with capsaicin, an agent shown to deplete of neuropeptides, had significantly reduced BBB permeability, cerebral oedema and functional deficits as compared vehicle-treated controls. These studies demonstrated that SP release, as part of neurogenic inflammation, was integrally linked to increased vascular permeability and cerebral oedema following TBI. Consistent with an important role of SP in TBI, clinical and experimental studies have reported adverse effects of ACE [82] or NEP inhibition, the enzymes which degrade SP, as this exaggerates the effects of released SP [83]. Indeed, ACE and NEP inhibitors have been found to increase plasma extravasation [74].

Apart from reports from our own research group [76,77,78,81,84,85,86,87,88,89,90], the study of neuropeptides in acute nervous system injury has been mainly confined to isolated reports in peripheral nerve injury [91], spinal cord injury [92] and brain ischaemia [75,93,94]. In contrast, neuropeptides have been extensively studied in the peripheral nervous system, as well as in asthma, dental pain and osteoarthritis [26].

### 4.4. Cerebral Ischemia

To date, few groups have investigated the role of SP in cerebral ischaemia [75,93,94], and apart from our own recent studies, none have characterised the role of neurogenic inflammation. Early studies reported that hypoxia of the rat carotid body increased SP release as a function of the severity of the hypoxic insult [95]. This finding suggested that SP release may be a tissue response to hypoxia/ischaemia. Consistent with this, capsaicin pre- or post-treatment was shown to confer protection from neonatal hypoxia-ischaemia injury with a reduction in infarct volume and apoptosis, in addition to improved vascular dynamics [96]. Moreover, capsazepine conferred protection from increases in microvascular permeability following cerebral ischaemia [97], suggesting that the TRPVI receptor was integral to this response. TRPV1 activation was subsequently shown to be involved in increased BBB and blood-spinal cord barrier permeability in the setting of peripheral nerve injury [98]. In clinical ischaemia, increased SP has been previously reported in the serum of patients with complete stroke and TIA [93]. 

**Figure 2:**
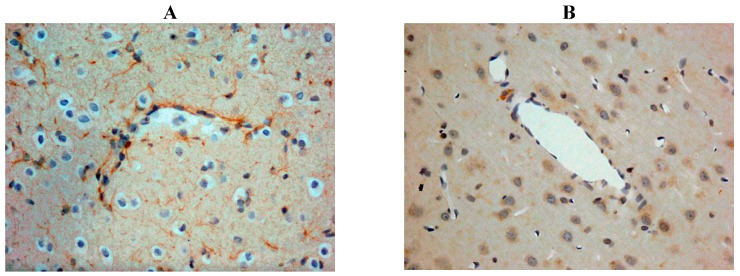
Increased SP immunreactivity was observed at 24h following acute ischaemic stroke (**A**), as compared to shams (**B**). This was particularly prominent in the perivascular tissue.

Our own rat studies have recently shown increased SP immunoreactivity within penumbral tissue at 24 h following stroke, being particularly marked in perivascular tissue (Figure 2). Such an increase in SP was confirmed through SP ELISA of the ischaemic hemisphere [87]. These findings were consistent with the increased SP levels previously reported in other central nervous system (CNS) disorders such as depression [99] and both experimental and clinical TBI [81,100]. The increase in SP was associated with marked disruption to the BBB, as evidenced by increased EB extravasation into the brain parenchyma at 24 h post-stroke, thus supporting previous observations of a delayed opening of the BBB [101]. At 24 h following 2 h middle cerebral artery occlusion with reperfusion, the increased BBB permeability was observed in the setting of profound cerebral oedema, suggesting that the oedema had a vasogenic component [87]. Furthermore, profound and persistent functional deficits with respect to motor, sensory and neurological function were observed [87]. 

## 5. NK1 Tachykinin Receptor Antagonists

Many groups have hypothesised that antagonists of tachykinin receptors may have several therapeutic applications and some of these have been tested in the settings of dental pain, neuropathic pain and migraine [26]. The antagonists have also been tested in a model of myocardial ischaemia/reperfusion combined with magnesium deficiency, where neurogenic inflammation was found to be an early event that initiated inflammatory and pro-oxidative processes that predispose the myocardium to reperfusion injury [102]. Inhibiting the SP-induced inflammation and pro-oxidative events with an NK1 tachykinin receptor antagonist (L-703, 606) reduced oxidative injury and improved functional recovery. 

**Figure 3:**
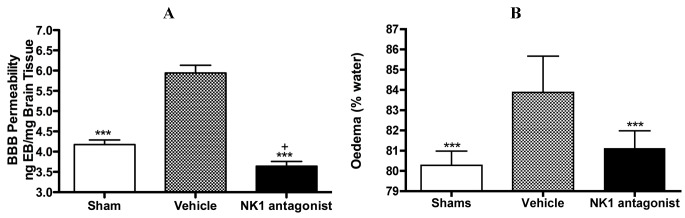
Marked BBB permeability, as measured by Evan’s Blue (EB) extravasation (**A**) and cerebral oedema, as measured by the wet weight-dry weight method (**B**) were observed at 24h following stroke. This was ameliorated by treatment with an NK1 tachykinin receptor antagonist. *** *p* < 0.001 compared to vehicle; ^+^ *p* < 0.05 compared to sham.

With respect to neurogenic inflammation, despite the potential utility of NK1 tachykinin receptor antagonists to reduce cerebral oedema, until recently only one group investigated the efficacy of NK1 tachykinin receptor antagonists following cerebral ischaemia [94]. This group reported over-expression of SP in conditions of cerebral ischaemia and speculated that SP may play a role in exacerbating ischaemic damage. They subsequently administered the NK1 tachykinin receptor antagonist SR140333 before the induction of stroke (i.c.v) and found that it significantly reduced infarct volume and improved neurological function as measured 24 h after focal cerebral ischaemia. Notably the antagonist was administered before injury induction and despite the positive findings, there has been no further work published in this area until our own recent studies. We sought to more thoroughly characterise the effects of NK1 tachykinin receptor inhibition following acute ischaemic stroke. In our studies (Figure 3) employing the rat thread model of stroke, we reported a significant improvement in BBB integrity and a reduction in cerebral oedema following treatment with an NK1 tachykinin receptor antagonist at 4 h post-stroke onset [87]. Animals administered with the NK1 tachykinin receptor antagonist also demonstrated a marked recovery of functional outcome with greatly improved motor, sensory and neurological function over the 7 days post-stroke assessment period. Furthermore, the NK1 tachykinin receptor antagonist was able to reduce mortality and the incidence of intracerebral haemorrhage following combined treatment with tissue plasminogen activator [88]. Taken together with our previous studies in TBI [77,81], these findings suggest that neurogenic inflammation is a feature of acute brain injury and is associated with alterations in microvascular permeability and the development of vasogenic oedema. Interventions that block or inhibit such neurogenic inflammation are beneficial to outcome, even when administered several hours after the acute event.

## 6. Classical Inflammation

The role of SP in classical inflammation has been extensively studied. It is well known that SP plays a role in the initiation and modulation of a number of inflammatory responses including leukocyte activation, endothelial cell adhesion molecule expression, cytokine production and mast cell activation amongsy many others [103] and all of these effects are mediated by the NK1 tachykinin receptor [104]. Moreover, blocking SP binding at the NK1 tachykinin receptor may attenuate inflammatory processes that are SP-mediated. For example, several studies have confirmed that astrocytes become “reactive” in response to SP following acute injury, inducing mitogenesis and the production of several soluble mediators, such as cytokines, prostaglandins and thromboxane derivatives [105,106]. Receptor binding sites for SP have been shown to increase on glia after neuronal injury, suggesting a potential role in the glial response to TBI. SP and the NK1 tachykinin receptor are also widely expressed by inflammatory cells, including neutrophils, monocytes, eosinophils, dendritic cells and activated T cells [23,28,107] suggesting a role in neuroimmunomodulation. Indeed, SP has pleiotropic actions in inflammation, with the capacity to stimulate the degranulation of mast cells, and stimulate cytokine release including that of interleukin (IL)-1β, tumour necrosis factor (TNF)-α [108] and IL-6 [71,109]. Furthermore, SP can modulate the chemotaxis of monocytes [110] and neutrophils [111], along with their aggregation, superoxide production [23], adherence to endothelium, lysosomal enzyme release and phagocytic activity by neutrophils [112,113]. In turn, the cytokines Il-1β, TNF-α and interferon (IFN)-γ can stimulate the release of SP by brain endothelial cells, further potentiating the inflammatory response. High levels of SP are found on the surface of rat brain endothelial cell cultures following cytokine stimulation [114]. Furthermore, the NK1 tachykinin receptor antagonist Spantide was shown to reduce permeability increases induced by IFN-γ and TNF-α, whilst also decreasing the expression of intercellular adhesion molecule-1 (ICAM-1) [115]. Such findings suggest that SP plays a major role in the regulation of cytokine-induced BBB damage during inflammatory processes in the CNS [114]. 

At the level of the endothelium SP is also involved in the expression of adhesion molecules. In a study of experimental autoimmune encephalomyelitis treatment with the NK1 tachykinin receptor antagonist CP-96,345 was associated with a decline in disease signs in addition to a marked reduction in the expression of the adhesion molecules ICAM-1 and vascular cell adhesion molecule-1 on the CNS endothelium [116]. Such data suggests that NK1 tachykinin receptor antagonists act to stabilize the BBB. SP also induces endothelial cells to produce nitric oxide (NO) [117], which has been implicated as an injury factor in stroke, as well as priming polymorphonuclear cells for oxidative metabolism (superoxide production) [118], thus providing a source of reactive oxygen species. NK1 tachykinin receptor antagonists have been shown to reduce pre-necrotic perivascular inflammatory infiltration, as well as circulating histamine, prostaglandin E2 and lipid peroxidation products [102]. Furthermore, SP binding to its NK1 tachykinin receptor in the CNS has been shown to directly induce a non-apoptotic form of programmed cell death in hippocampal, striatal and cortical neurons that is independent of caspase activation, but still requiring gene expression [119].

The relationship between SP and mast cells in the setting of ischaemic stroke is particularly important. Mast cells release nerve growth factor which stimulates a rapid and large release of SP and CGRP. SP and mast cells act in a positive feedback loop whereby SP is able to significantly increase the number and activation of mast cells. Indeed, aside from their utility in blocking the deleterious effects of SP in the acute phase of injury following ischaemic stroke, NK1 tachykinin receptor antagonists may also have a utility in the pathogenesis of stroke. A number of studies have now detailed a role for SP in thrombosis and atherosclerosis, specifically in the regulation of platelet function [120]. Specifically, SP has been shown to influence platelet function by acting as a secondary platelet agonist [121]. Administration of the NK1 tachykinin receptor antagonist L733-660 was shown to reduce thrombus generation in vitro under arterial flow condition, increase bleeding time in mice and provide protection against thromboembolism [122]. Furthermore, SP has been shown to induce plaque destabilisation [123]. Through mast cell activation, SP promotes mast cell-dependent intra-plaque haemorrhage, [123]. These findings suggest that SP promotes mast cell-dependent plaque destabilisation and provides a direct link between tachykinins and vascular inflammation. This may be particularly important when considering stroke pathophysiology. Both of these actions were prevented with treatment with the NK1 tachykinin receptor antagonist Spantide. NK1 tachykinin receptor antagonists may therefore offer an alternative to current anti-thrombotic agents, with a better safety profile [120,121].

## 7. Conclusions

Neurogenic inflammation has long been known to cause plasma extravasation and swelling in peripheral tissues. Only recently has the concept of neurogenic inflammation been extended to the brain with studies of stroke and traumatic brain injury demonstrating that perivascular SP is increased following acute injury to the brain and that it is associated with marked disruption to the BBB and the development of vasogenic oedema. Subsequent intervention studies have documented the efficacy of NK1 tachykinin receptor antagonists in ameliorating such adverse events. Therefore, modulation of neurogenic inflammation through inhibition of the SP pathway using NK1 tachykinin receptor antagonists may provide a novel approach to the management of cerebral oedema following stroke and other forms of acute brain injury.
